# Antioxidant Effect of a Plant-Derived Extracellular Vesicles’ Mix on Human Skin Fibroblasts: Induction of a Reparative Process

**DOI:** 10.3390/antiox13111373

**Published:** 2024-11-09

**Authors:** Rossella Di Raimo, Davide Mizzoni, Antonella Aloi, Giulia Pietrangelo, Vincenza Dolo, Giuseppina Poppa, Stefano Fais, Mariantonia Logozzi

**Affiliations:** 1 ExoLab Italia, Tecnopolo D’Abruzzo, 67100 L’Aquila, Italy; rossella@exolabitalia.com (R.D.R.); davide@exolabitalia.com (D.M.); antonella.aloi@exolabitalia.com (A.A.); giulia.pietrangelo@exolabitalia.com (G.P.); 2 Department of Oncology and Molecular Medicine, Istituto Superiore di Sanità, 00161 Rome, Italy; 3 Department of Clinical Medicine, Public Health, Life and Environmental Sciences, University of L’Aquila, 67100 L’Aquila, Italy; vincenza.dolo@univaq.it (V.D.); giuseppina.poppa@graduate.univaq.it (G.P.)

**Keywords:** plant-derived extracellular vesicles, antioxidants, natural bioactives, anti-aging, skin repair, fibroblasts sirtuin, vimentin, wound repair

## Abstract

Plant-Derived Extracellular Vesicles extracellular vesicles (PDEVs) from organic agriculture (without the use of pesticides and microbicides) contain high levels of antioxidants. Organic PDEVs have shown an increased antioxidant power compared to PDEVs from single plants, suggesting a synergistic effect of the bioactives constitutively expressed in the PDEVs from single fruits. With this study, we wanted to investigate the beneficial effects of a mix of PDEVs on human skin cells. We found detectable levels of citric acid, ascorbic acid, glutathione, catalase, and SOD in a mix of PDEVs deriving from five different fruits (grape, red orange, papaya, pomegranate, and tangerine). We then treated H_2_O_2_-conditioned fibroblasts with the mix of PDEVs. The results showed that the PDEVs’ mixture reverted the H_2_O_2_-induced redox imbalance, restoring mitochondrial homeostasis, with a strong reduction of mitochondrial anion superoxide and an increase in sirtuin levels. The antioxidant action was consistent with wound repair on a lesion produced in a fibroblast’s monolayer. This result was consistent with an increased level of vimentin and matrix metalloproteinase-9, whose expression is directly related to the efficiency of the reparative processes. These data support a beneficial role of PDEVs in both preventing and treating skin injuries through their potent antioxidant and reparative activities.

## 1. Introduction

Skin is the largest organ in our body, representing the most important barrier against external stimuli, such as pollution and UV radiation [1,2]. The most important function of stratum corneum is the protection against dryness; in fact, impairments of skin integrity led to an increase in trans-epidermal water loss and consequent insufficient skin moisture [3,4,5]. As widely demonstrated, UV can induce oxidative stress in human skin cells, generating an increased production of reactive oxidative species that can in turn affect lipid integrity, reach the genetic material in cell nuclei, and trigger DNA damage, with consequent impairment of cellular function and cell death. Skin aging is a complex mechanism due to both intrinsic and extrinsic factors. However, evidence supports that redox imbalance plays a key role in the prime cause of skin aging. Many environmental factors take part in the skin aging, including UV exposure and pollution [6,7,8].

Mitochondria are fundamental in the skin’s protection against inflammation and local ROS, mostly through the production of collagen and elastin. Mitochondria are considered the powerhouse of cells. In fact, they generate energy in the form of ATP [9], which involves a series of enzyme complexes and transporters. Mitochondrial membrane potential derives from redox transformation and represents a key checkpoint involved in the production of energy within cells [10]. Mitochondrial membrane potential is considered a valuable indicator of mitochondria’s health, inasmuch as it maintains an active control of the ions’ transport [10,11,12]. Actually, mitochondrial membrane potential is not a stable value, and the oxidative stress is directly involved in the impairment of the mitochondrial membrane potential [10,13,14]. Mitochondria generate 90% of ROS at the cellular level, where the antioxidant molecules are crucial to maintain redox balance within the cells in order to avoid serious cell damage [15,16]. In particular, mitochondrial superoxide anion is normally converted in oxygen and water by cellular antioxidant systems, but in pathological conditions it may overcome the antioxidant apparatus, leading to different levels of cellular damage [17,18]. During the oxidative stress and inflammation, while passing through a mitochondrial dysfunction, different proteins are involved, including sirtuin 1. Sirtuin 1, probably the most known of the sirtuins’ family, is a NAD^+^-dependent deacetylase involved in some regulatory pathways often leading to chromatin silencing and a general decrease of the energetic state of the cell [19]. Sirtuin also participates in the regulation of several proteins involved in age-related processes at the transcriptional level [20]. It was also shown that the age-related decrease of sirtuin 1 content is associated with a significant inhibition of cellular proliferation [21,22,23]. Natural compounds, such as resveratrol, exert beneficial effects in skin-derived cells by increasing sirtuin 1 expression [24,25,26,27,28,29,30].

There is clear evidence that plants contain different antioxidants that have been used in treating different pathological conditions caused by redox imbalances, including skin damages and tumors [31,32,33,34,35,36,37]. More recently, the beneficial effect on skin health of extracellular vesicles isolated from plant extracts (PDEVs) has been widely reported. In fact, PDEVs contain a variety of antioxidants, with both enzymatic that non-enzymatic activity, that efficiently reduce damages induced by oxidative stress and help wound healing in treated cells. In this regard, increased ROS at the skin level, leads to decreased proliferation of both fibroblasts and keratinocytes, with a reduced skin cell turnover and decreased production of elastin [8,38,39].

Wound healing is a dynamic skin process that involves different events such as inflammation, proliferation and migration of different cell types, such as fibroblasts [40,41,42,43,44,45,46]. In this process are involved some proteins, such as matrix metalloproteinase and vimentin, that drive cellular proliferation and migration to the wound site [47,48,49,50,51,52,53,54,55,56].

The antioxidant activity of PDEVs may have a role in wound healing by directly influencing both gene expression and/or intracellular pathways that are known to be involved in this process. For example, extracellular vesicles from wheat and grapefruit increase collagen production along with fibroblasts proliferation and migration to the wound site. Moreover, exosomes promote the formation of tube-like structures involved in the angiogenetic processes [57,58,59].

All in all the antioxidants constitutively contained in PDEVs can efficiently contrast damages related to skin aging, reducing intracellular ROS levels and stimulating collagen production [39,60,61,62,63,64,65].

With the above background we decided to test the effect of a PDEVs’ mix, as represented by a mix fruits, on human skin fibroblasts in different culture conditions, in order to assess the possible use of this PDEVs’ mix as a skin-aging reversing agent.

## 2. Materials and Methods

### 2.1. Fruit Material

Tangerines (*Citrus reticulata*), blood oranges (*Citrus sinensis* ‘Blood Orange’), papayas (*Carica papaya* L.), pomegranates (*Punica granatum*), and grapes (*Vitis vinifera*) were purchased from several Italian farms with organic farming certifications. After washing with water and bicarbonate, the fruits were peeled for the extraction of the juice through a juice extractor. Juices were stored at −80 °C for subsequent analysis.

### 2.2. Extracellular Vesicles Isolation

After mixing the fruit extracts, samples were centrifuged at 500× *g* for 10 min; the supernatants were filtered with 100 µm filters and serially centrifugated at 2000× *g* for 20 min to eliminate cell debris and then at 15,000× *g* for 30 min to collect the fraction enriched in microvesicles. The supernatants were subsequently ultracentrifuged in a Sorvall WX Ultracentrifuge Series (Thermo Fisher Scientific, Waltham, MA, USA) at 110,000× *g* for 1 h 30 min to collect the nanovesicles. The pellet was resuspended in an appropriate buffer for downstream analyses [35,66].

### 2.3. Ferric Reducing Antioxidant Power (FRAP) Assay Kit

The Ferric Reducing Antioxidant Power (MAK369, Sigma-Aldrich, St. Louis, MO, USA) Assay Kit provides a quick, sensitive, and easy way for measuring the antioxidant capacity of various biological samples, using antioxidants as reductants in a redox-linked colorimetric reduction wherein Fe^3+^ is reduced to Fe^2+^. Samples were added in a 96-well plate and mixed with the Rection Mix (Reaction Mix is composed of a FRAP Assay Buffer, an FeCl_3_ Solution, and a FRAP Probe). The optical densities were read at 594 nm.

### 2.4. Ascorbic Acid Assay

The Ascorbic Acid Assay Kit (Sigma-Aldrich, USA) was used to quantify the ascorbic acid concentration in PDEVs. Appropriate dilutions of PDEVs were plated into a 96-well plate; subsequently, a catalyst and then a reaction composed of ascorbic acid buffer, ascorbic acid probe, and ascorbic acid enzyme mix were added to each well. After 5 min of incubation, fluorescence was read in a microplate reader (Promega, Madison, WI, USA) at Ex/Em = 535/587 nm.

### 2.5. ATP Assay Kit

An ATP Assay Kit (Colorimetric) (Abcam, Cambridge, UK) was used to measure total ATP in PDEVs. Briefly, the assay quantifies a product from the phosphorylation of glycerol. Standards and samples at the optimal dilution were added in a 96-well plate, then the reaction mix was added to each standard and sample well, and after a 30 min incubation, the optical densities were measured using a microplate reader at 570 nm.

### 2.6. Catalase Activity Assay

In fruit-derived nanovesicles, Catalase activity was measured using the Catalase Activity Assay (Abcam, Cambridge, UK), a fluorometric kit for detection and quantification Catalase. The test was performed following the manufacturer’s instructions, and after the developer addiction, fluorescence was read at Ex/Em = 535/587 nm using a microplate reader (Promega, Madison, WI, USA). One unit of catalase corresponds to the amount of catalase that will decompose 1 µmol of H_2_O_2_ per minute at pH 4.5 at 25 °C.

### 2.7. Citric Acid Assay

The detection and quantification of citric acid was performed in skin cells regeneration using a Citric Acid (CA) Colorimetric Assay Kit (MBS2563963, MyBioSource, San Diego, CA, USA).

The samples were incubated with the reaction reagents for 30 min at room temperature, and then the reaction product was analyzed at a wavelength of 545 nm.

### 2.8. Reduced Glutathione (GSH) Detection and Quantification Assay

Reduced glutathione (GSH) levels in PDEVs were detected and quantified through a colorimetric assay, The Glutathione Colorimetric Detection Kit (Thermo Fisher, USA). Samples were incubated for 20 min at room temperature with detection reagent and reaction mixture (NADPH and glutathione reductase), and then the optical densities were recorded at 405 nm.

### 2.9. Superoxide Dismutase (SOD) Activity Assay

To quantify the activity of the superoxide dismutase, a colorimetric assay (Superoxide Dismutase Activity Kit, Thermo Fisher, USA) was used. Briefly, PDEVs’ preparations were loaded in a 96-well plate with the substrate, then the chromogenic detection reagent was added to each sample, and after 20 min’ incubation at room temperature, the optical densities were recorded at 450 nm.

### 2.10. Nanoparticle Tracking Analysis

Size distribution and concentration of extracellular vesicles in liquid suspension were analyzed through Nanoparticle Tracking Analysis (NTA) from Malvern (NanoSight NS300, Salisbury, UK). For each measurement, five videos of typically 60 s duration were taken, and data were analyzed using the NTA 3.4 software (Malvern Instruments, Malvern, UK). The Brownian motion of each particle was tracked using the Stokes–Einstein equation: D° = kT/6πηr, where D° is the diffusion coefficient, kT/6πηr = f0 is the frictional coefficient of the particle, for the special case of a spherical particle of radius r moving at a uniform velocity in a continuous fluid of viscosity η, k is Boltzmann’s constant, and T is the absolute temperature.

### 2.11. Dynamic Light Scattering

Dynamic light scattering (DLS) (Malvern Instrument, UK) was used for the evaluation of the zeta potential of plant-derived extracellular vesicles. Evaluation of zeta potential allows prediction of the stability or electrostatic interactions of extracellular vesicles in dispersions. 

Data were analyzed using ZS XPLORER software (Software version: 1.3 14.7, Malvern Instrument, UK).

### 2.12. Transmission Electron Microscopy (TEM)

The isolated PDEVs were resuspended in PBS and, after a proper dilution, placed on 200-mesh carbon-coated copper grids. PDEVs were let adhere to the grids and fixed with glutaraldehyde 2% (Electron Microscopy Sciences, Hatfield, PA, USA) in PBS. Then, 3 washing steps with milliQ water were performed, followed by negative staining with a phosphotungstic acid 2% solution. TEM images were captured using a Philips CM 100 Electron Microscope [67,68,69,70].

### 2.13. Cell Line

Normal human dermal fibroblast cells (NHDFs) (Sigma-Aldrich, USA) isolated from the dermis of adult skin where cultured in Low-serum cell culture medium (Fibroblast Growth Medium 2, Sigma-Aldrich, USA) supplemented with antibiotics and antimycotics in incubator with an atmosphere of 5% CO_2_. For each *in vitro* experiments we used 10^9^ PDEVs.

### 2.14. Staining Protocol of PDEVs with a Fluorescent Probe (Dil 1,1′-Dioctadecyl-3,3,3′,3′-tetramethylindocarbocyanine Perchlorate)

To study cellular uptake of PDEVs, vesicles were marked with Dil, a fluorescent lipophilic cationic indocarbocyanine dye widely used to label membrane lipid bilayer (1,1′-Dioctadecyl-3,3,3′,3′-tetramethylindocarbocyanine perchlorate, 42364, Sigma-Aldrich, USA).

A stock solution of Dil was prepared, dissolving the power in DMSO and then incubating the extracellular vesicles with a 5 µM dye solution for 30 min at 37 °C. After incubation, the vesicles were ultracentrifugated at 110,000 rpm for one hour to remove the unlabeled dye, and labeled-PDEVs were used for cellular treatment.

Fibroblasts (NHDF) were treated with dil-labeled PDEVs, and after 24 h, 48 h, and 72 h of treatment, cells were fixed with paraformaldehyde (4%). The slides were counterstained with DAPI (DAPI/Antifade solution, Sigma-Aldrich, USA), and images were acquired with the Optika microscope (IM-5FLD, Optika Microscopes, Bergamo, Italy). Mean intensity of fluorescence was measured using ImageJ software (v1.53t, National Institutes of Health of the United States).

### 2.15. Mitochondrial Membrane Potential Measurement

To analyze the mitochondrial membrane potential, MitoTracker^®^ Dyes for mitochondria labeling (Thermo Fisher, USA), was used. This green-fluorescent dye passively diffused across plasma membrane and localized in active mitochondria; the reduced probes do not fluoresce until they enter in live cells, where they are oxidized to the corresponding fluorescent mitochondrion-selective probe and then sequestered in the mitochondria. After the induction of oxidative stress (500 µM H_2_O_2_), fibroblasts were incubated with prewarmed MitoTracker^®^ probe staining solution at 37 °C and 5% CO_2_ for 30 min. After incubation cells were centrifugated and resuspended in a fresh prewarmed medium or buffer. Green fluorescence was read at Ex/Em = 490/516 using a fluorescence microplate reader. Data are expressed as mean intensity of fluorescence.

### 2.16. Mitochondrial Superoxide Assay

To evaluate the antioxidant effect of PDEVs, NHDF were treated with 500 µM H_2_O_2_ for 24 h to induce oxidative damage and then with PDEVs. After 24 h treatment, mitochondrial superoxide was measured in cells treated with PDEVs and control untreated cells using the Mitochondrial Superoxide Detection Kit (Abcam, Cambridge, UK). This assay is composed of the MitoROS 580 dye that reacts with the mitochondrial superoxide present in live cells to generate a red fluorescence signal at Ex/Em = 540/590 nm. Mean intensity of fluorescence was measured using a fluorescence microplate reader. Data are expressed as M.I.F. (a.u.).

### 2.17. Sirtuin Quantification

Detection and quantification of sirtuin in PDEVs were performed using an ELISA Kit: SIRT1 (sirtuin1) Human SimpleStep ELISA^®^ Kit (ab171573, Abcam, UK). Samples and standards are added to the wells and incubated with the antibody mix. After washing to remove unbound material, TMB substrate is added to wells and incubated to allow the catalysis by HRP, generating a blue coloration. Finally, the stop solution is added to the wells and the optical densities are read at 450 nm.

### 2.18. Wound Healing Assay

Fibroblast cells (NHDF) were seeded in order to have a monolayer. Once the monolayer was formed, we made a scratch using a micropipette tip to mimic a wound. The cells were then treated with PDEVs, and images were acquired at time 0, after 24 h, and 48 h treatment using the Optika microscope (IM-5FLD, Optika Microscopes Italy). Wound gaps were measured using the PROview software (Software version: x64, Optika Microscopes Italy).

### 2.19. Collagen I Evaluation

To evaluate collagen I expression after PDEV treatment, we seeded fibroblasts on coverslips, then we induced a scratch and treated cells with PDEVs. Briefly, cells after treatment were fixed with paraformaldehyde (4%) and permeabilized with Triton X solution (0.1%). After incubation of blocking solution, cells were incubated with rabbit primary collagen-binding antibody I (ab34710, Abcam, UK). The next day, goat anti-rabbit secondary antibody Alexa Fluor^®^ 488 (ab150077, Abcam, UK) was added. And the samples were counterstained with DAPI (DAPI/Antifade solution, Sigma-Aldrich, S7113). Images were acquired with the Optika microscope (IM-5FLD, Optika Microscopes Italy), and the fluorescence intensity was measured using ImageJ software (Sofware version: v1.53t, National Institutes of Health of the United States).

### 2.20. Matrix Metalloproteinase-9 Evaluation

As described above, we induced a scratch in the monolayer of fibroblasts seeded on coverslips. After 24 h treatment, cells were fixed with paraformaldehyde (4%) and stained with monoclonal anti-Mmp9—FITC (SAB5200306, Sigma-Aldrich, USA). The slides were counterstained with DAPI (DAPI/Antifade solution, Sigma-Aldrich, USA), and images were acquired with the Optika microscope (IM-5FLD, Optika Microscopes Italy). Mean intensity of fluorescence was measured using ImageJ software (Sofware version: v1.53t, National Institutes of Health of the United States).

### 2.21. Vimentin Quantification

As described above (Section 2.18), we induced a scratch in the monolayer of fibroblasts seeded on 24-well plates. After a 24 h treatment with PDEVs, extracellular vimentin was measured using VIM (Human) ELISA Kit (KA3127, Novus Biologicals, Abingdon, UK). Anti-vimentin antibody was pre-coated onto 96-well plates, then standards, test samples, and anti-vimentin HRP conjugated antibody were added to the wells. After incubation, wells were washed in order to eliminate unbound conjugates. TMB substrates (A and B) were added to wells, and they were catalyzed by HRP to produce a blue-colored product that changed into yellow after adding an acidic stop solution. The density of yellow is proportional to the vimentin amount of sample captured in plate. The O.D. absorbance was read at 450 nm in a microplate reader.

### 2.22. Statistical Analysis

Results are reported as the means ± standard error (SE), and calculations were performed using GraphPad Prism software (Software versione 8.0.2, USA). An unpaired *t*-test (Student’s *t*-test) and one-way ANOVA Bonferroni were applied to analyze the results. Statistical significance was set at *p* < 0.05.

## 3. Results

### 3.1. Characterization of the PDEVs Mix

#### 3.1.1. Bioactives’ Content

We have first characterized the content in bioactives of a mix of different fruits. In our experiment, we exploited a combination of PDEVs isolated from grape (*Vitis vinifera*), pomegranate (*Punica granatum*), orange (*Citrus sinensis*), tangerine (*Citrus reticulata*), and papaya (*Carica papaya*). The mix of these fruits has shown a very high level of bioactive compounds. PDEVs were characterized through different colorimetric kits to detect and quantify their antioxidant cargo. The summarized results obtained from the analysis of 10^9^ PDEVs can be found in Table 1.

#### 3.1.2. Size Distribution and Zeta Potential Analysis

**Figure 1:**
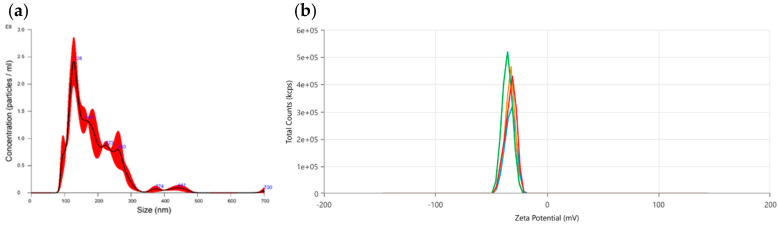
PDEVs biophysical characterization. (**a**) Size and distribution of PDEVs through NTA; (**b**) Distribution of PDEVs’ zeta potential.

Then we characterized through Nanoparticle Tracking Analysis the PDEVs’ mix in order to establish their concentration and size distribution. As reported in Figure 1a, PDEVs isolated from the above-mentioned mix of fruit extracts have the typical size distribution of extracellular vesicles, with size equal to 189.5 ± 2.1 nm. Moreover, in order to further characterize PDEVs isolated from fruits, we used the Zetasizer to analyze the zeta potential in a liquid suspension. The zeta potential measured was −33.68 ± 1.3 mV, and the results are reported in Figure 1b.

#### 3.1.3. Morphological Characterization

**Figure 2:**
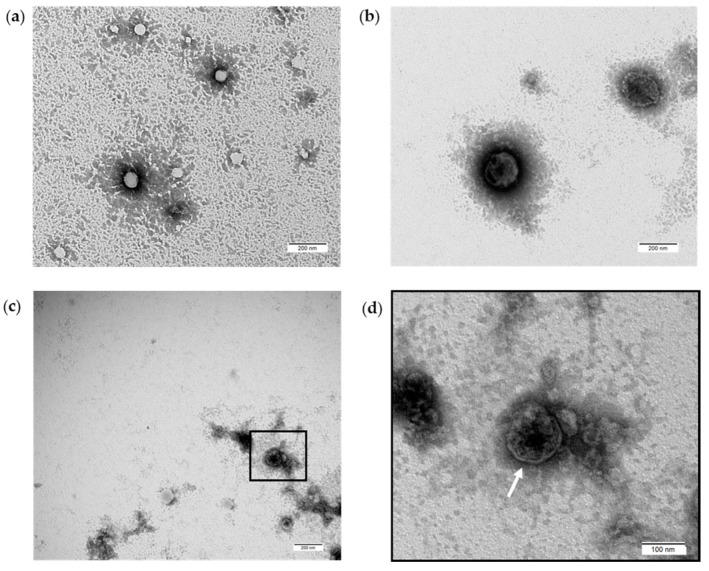
PDEVs morphological characterization through TEM. (**a**) Round structure and membrane integrity of PDEVs with sizes between 50 and 80 nm; (**b**) Round structure and membrane integrity of PDEVs with sizes between 150 and 200 nm; (**c**) PDEVs plasma membrane visible at 34.000 magnification and (**d**) 64.000 (insert). The arrow indicates the plasma membrane of vesicles.

PDEVs were also characterized by Transmission Electron Microscopy (TEM) to better evaluate their PDEVs-like morphology [71]. In fact, this analysis, due to its nanometer resolution, can efficiently distinguish Extracellular Vesicles from non-EV particles. The results showed that the examined samples contained vesicles with a typical round shape that did not show any sign of damage from a morphological point of view; they also had a size ranging from 50–80 nm (Figure 2a) to 150–200 nm (Figure 2b), suggesting their EV-like nature. TEM analysis also confirmed the presence of an intact bilayer membrane, witnessed by a lighter area that surrounds the black electron-dense vesicles’ content (Figure 2). Thus, our PDEVs contained a heterogeneous population in size, round in shape, and enclosed by a membrane. For negative staining, the plasma membrane appears as a white filament surrounded by a dark background indicated by an arrow and clearly visible in Figure 2c,d.

### 3.2. PDEVs Uploading into Human Skin Fibroblasts

In order to track the uptake of extracellular vesicles over time, we labeled extracellular vesicles with Dil, an orange-red lipophilic dye. This dye is usually weakly fluorescent until incorporated into membranes. The stain with lipophilic dye is usually used to label extracellular vesicles and trace their distribution in *in vitro* and *in vivo* cells [72,73,74,75,76,77].

**Figure 3:**
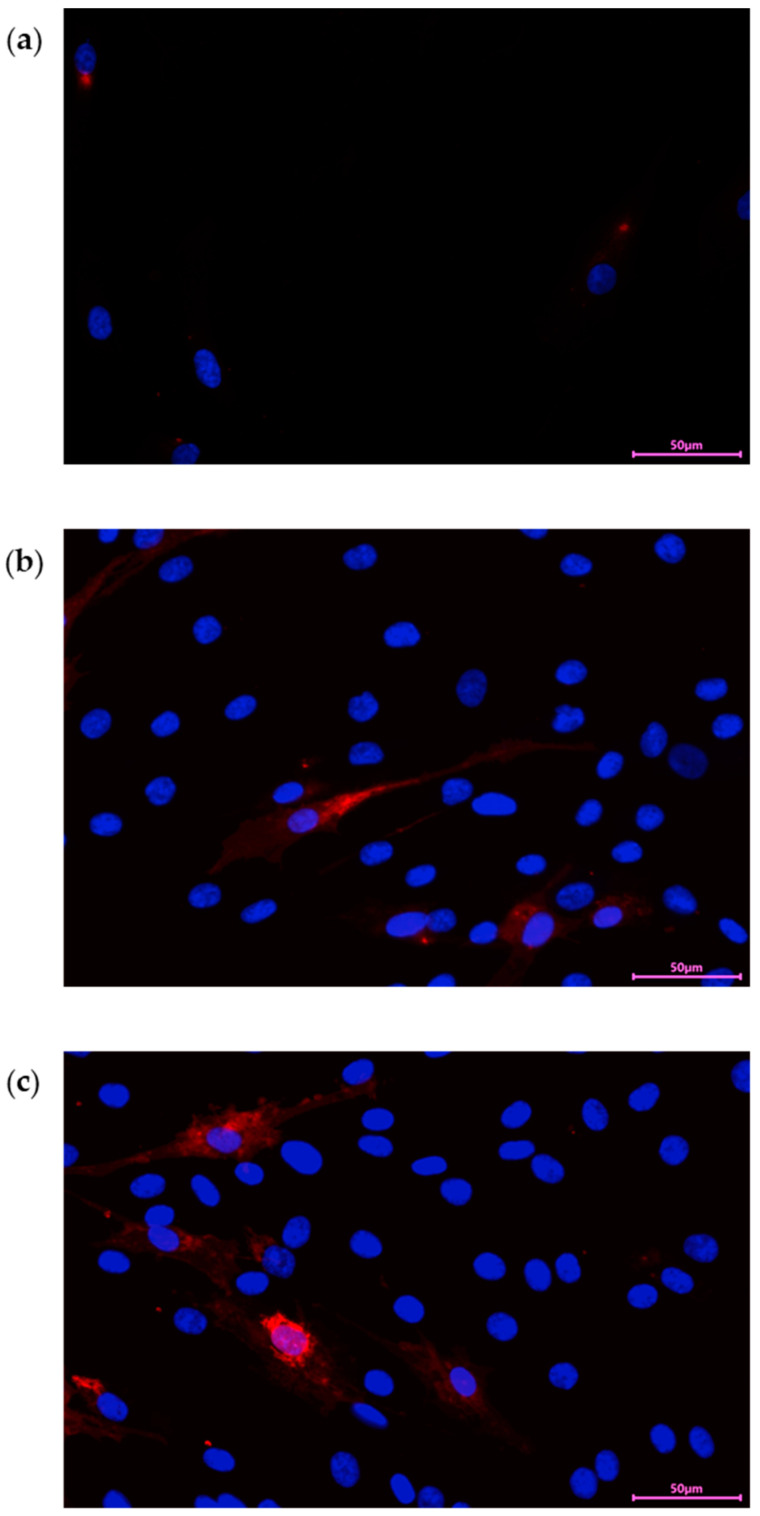
Cellular uptake of Dil-PDEVs in skin fibroblasts after (**a**) 24 h of treatment; (**b**) 48 h of treatment; and (**c**) 72 h of treatment. PDEVs were labeled with Dil (red), and nuclei were counterstained with DAPI (blue).

In our experiment, we stained PDEVs with Dil for 20 min at 37 °C, then we washed unincorporated dye and used PDEVs-Dil in cell treatment for different time points. Data reported in Figure 3 show that PDEVs were efficiently internalized in cells after 24 h (Figure 3a) and that fluorescent signal was directly proportional to treatment time. Moreover, results demonstrated that PDEVs can efficiently reach the cell nucleus, where they may affect the genome (Figure 3c).

### 3.3. PDEVs Counteract Oxidation-Induced Damage with Hydrogen Peroxide

#### 3.3.1. Effect on Mitochondrial Metabolism

**Figure 4:**
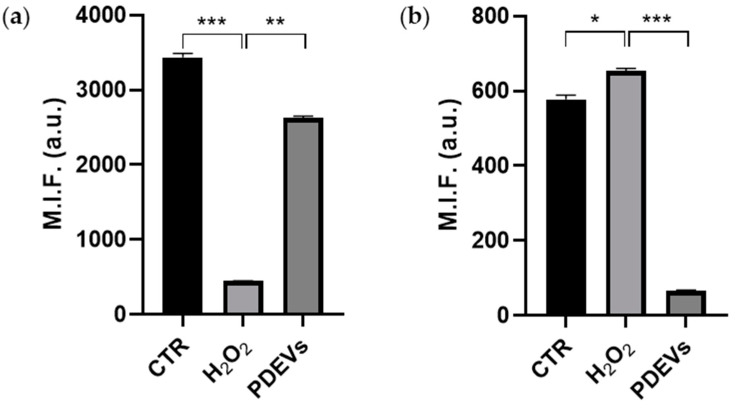
PDEVs effect of mitochondrial metabolism. (**a**) Analysis of mitochondrial membrane potential; (**b**) analysis of mitochondrial anion superoxide levels. Data are expressed as mean ± SE. * *p* < 0.05, ** *p* < 0.01, and *** *p* < 0.001. Statistical analysis was performed using one-way ANOVA Bonferroni. M.I.F. (a.u.) = Mean I Intensity of Fluorescence (arbitrary unit).

For the reasons reported in the introduction section, we wanted to investigate how PDEVs can mitigate progressive aging using a model of fibroblasts treated with H_2_O_2_ [78,79]. To evaluate the effect of PDEVs in cellular metabolism, we induced an oxidative stress in *in vitro* fibroblasts through H_2_O_2_ treatment, mimicking an effect on the cell’s senescence (Supplementary Figure S1), and then we treated cells with the PDEVs’ mix. As shown in Figure 4a, PDEVs can restore unbalanced mitochondrial membrane potential induced by oxidative stress. H_2_O_2_ treatment significantly reduced mitochondrial membrane potential (447 ± 3 M.I.F.; *p* < 0.001) compared to control cells (3434 ± 56 M.I.F.), whereas cells subjected to oxidation and then treated with PDEVs have an increased potential level (2626 ± 27 M.I.F.; *p* < 0.01) compared to the H_2_O_2_-treated fibroblasts.

In the following series of experiments, we also evaluated the mitochondrial superoxide anion levels in untreated cells, either cells undergone H_2_O_2_ or cells treated with PDEVs following H_2_O_2_. In the H_2_O_2_-treated cells, we measured a 13% increase in mitochondrial superoxide anion compared to control cells (*p* < 0.05), with values equal to 578 ± 11 M.I.F. (a.u.) of untreated cells and 654 ± 6 M.I.F. (a.u.) of oxidated cells. The most significant result was observed in fibroblasts subjected to oxidation and then treated with Extracellular Vesicles. Indeed, as shown in Figure 4b, a significant reduction (*p* < 0.001) in superoxide anion levels was observed in the samples treated with PDEVs’ mix (65 ± 2 M.I.F.) compared to oxidated cells; in particular, the anion value is 10-fold lower in PDEV-treated samples compared to human fibroblasts undergone H_2_O_2_ treatment. We also obtained data demonstrating that in non-oxidated conditions, PDEV treatment efficiently reduces mitochondrial superoxide anion in fibroblasts’ cultures.

#### 3.3.2. Effects of the PDEVs’ Mix on Some Aging-Related Molecules

**Figure 5:**
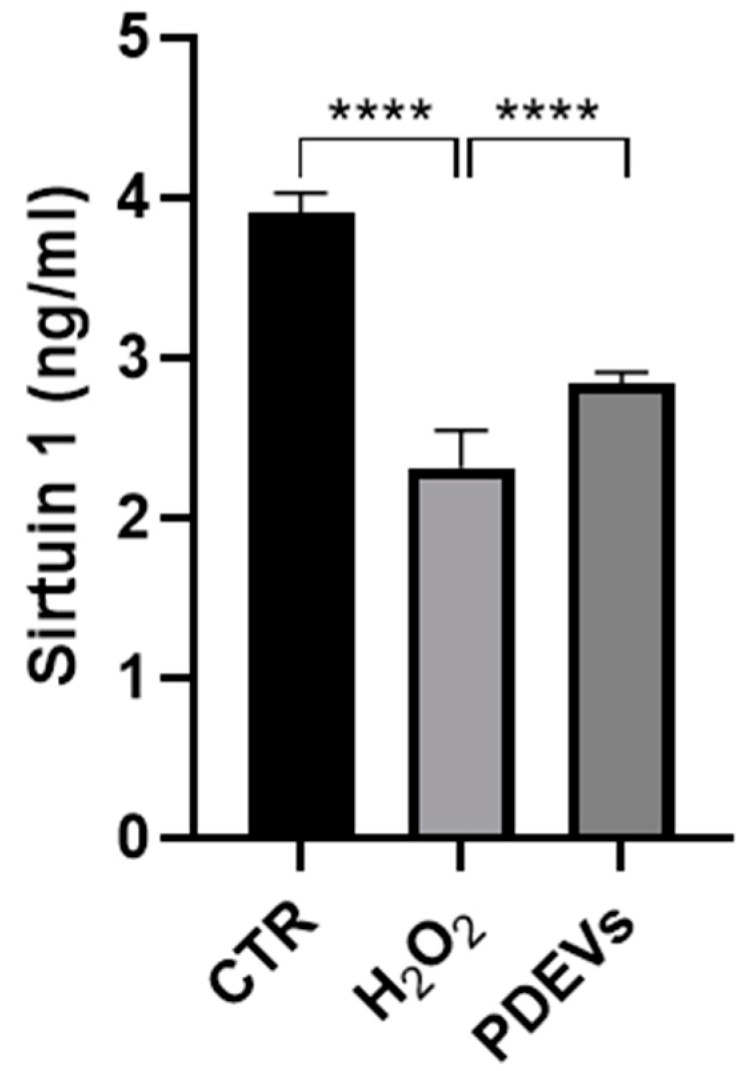
Quantification of extracellular sirtuin 1 concentration. Data are expressed as mean ± SE. **** *p* < 0.0001. Statistical analysis was performed using one-way ANOVA Bonferroni.

Sirtuin 1 is the most extensively studied member of the sirtuin family that is involved in oxidative stress response, inflammation, and mitochondrial functions, as well as in the transcriptional regulation of several proteins involved in age-related processes [20]. We thus quantified the concentration of sirtuin 1 in cell culture supernatants of human fibroblasts treated with either H_2_O_2_ or H_2_O_2_ + PDEVs or left untreated. As reported in Figure 5, extracellular sirtuin 1 concentration is significantly reduced after oxidation (2.32 ± 0.03 ng/mL; CTR 3.91 ± 0.07 ng/mL; *p* < 0.0001), while the treatment of oxidated skin fibroblasts with the PDEVs’ mix efficiently increased sirtuin 1 levels as compared to cells treated with H_2_O_2_ only (2.84 ± 0.02 ng/mL; *p* < 0.0001).

These data showed that the PDEVs’ mix significantly restored sirtuin 1 levels in skin-derived human fibroblasts after oxidative stress, while not reaching the sirtuin 1 levels detected in the control cells. This was not to cytotoxic effect inasmuch as it occurred without any evidence of a cytotoxic effect. This set of results supports the beneficial effect of PDEVs’ mix in mitigating progressive skin aging.

### 3.4. PDEVs Promote Regenerative Processes After Wound Induction

#### 3.4.1. PDEVs Promote Wound Closure

**Figure 6:**
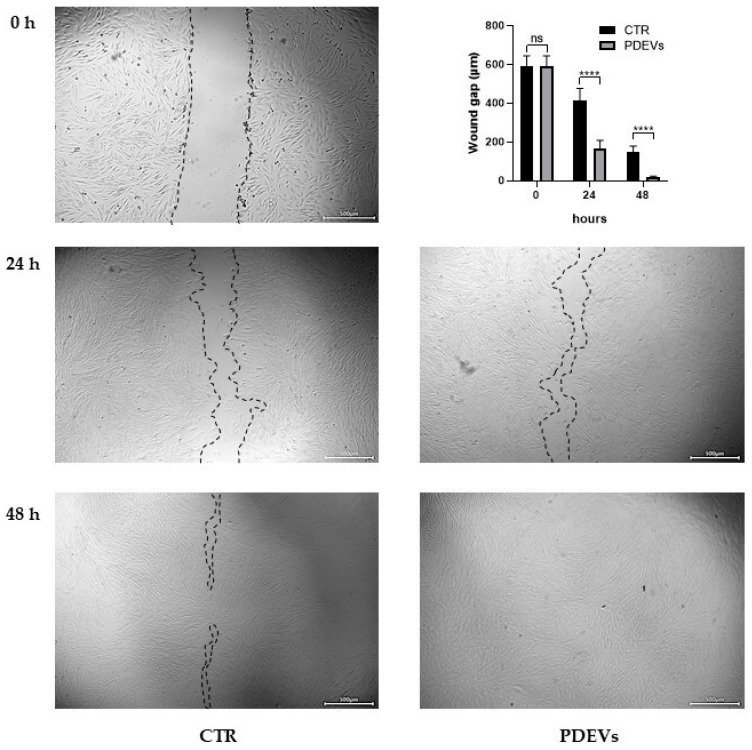
Wound healing assay of skin fibroblasts. Representative images are shown from three independent experiments. Data are expressed as mean ± SE. ns: not significant; **** *p* < 0.0001. Statistical analysis was performed using unpaired *t*-test (Student’s *t*-test). Scale bar = 500 µm.

Fibroblasts play a crucial role in skin repair. We thus used this cellular model to investigate the effects of our PDEVs’ mix on the wound healing after the induction of a scratch in the fibroblasts’ monolayer. Fibroblasts were seeded in a 24-cell plate in order to have a uniform monolayer of cells; then we induced an injury in the monolayer and treated them with PDEVs. Images of untreated control and treated fibroblasts were acquired after 24 and 48 h. For *in vitro* evaluation of wound closure, we measured the distance between the two cell fronts. The results are summarized in Figure 6. Our experiments have shown that the PDEVs’ mix significantly speeded up wound healing at both 24 (*p* < 0.0001) and 48 h (*p* < 0.0001) by reducing the distances between cells’ fronts. As shown in Figure 6, the wound gap at 24 h in control cells was 412 ± 21 µm, while after the PDEVs’ mix treatment it was reduced to 165 ± 14 µm. Similarly, after 48 h, the distance between the two cells’ fronts was 148 ± 10 µm in untreated control cells compared to 16 ± 3 µm in PDEV-treated cells, with an almost complete closure of the scratch after PDEVs’ mix treatment.

#### 3.4.2. PDEVs Increase Collagen I Expression After Injury Induction

**Figure 7:**
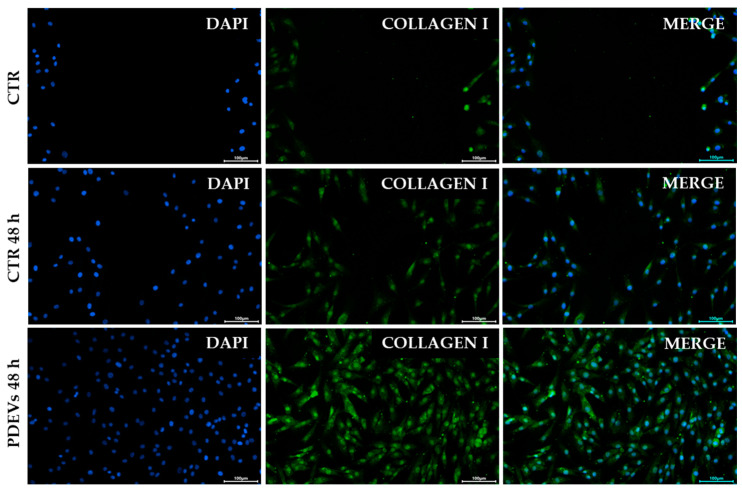
Collagen I expression in skin fibroblasts at the wound site. Cells were stained with anti-collagen I primary antibody, subsequently a secondary antibody AlexaFluor^®^ 488 conjugated was added (green) and nuclei were counterstained with DAPI (blue). Scale bar = 100 µm.

Collagen is the most abundant protein in the human body, particularly expressed in connective tissues such as cartilage and bone. Collagen makes up 90% of our skin, where it plays a crucial role in dermal structure, working in synergy with other proteins in the extracellular matrix. Collagen helps to consolidate the skin’s structure, imparting firmness, elasticity, and resistance to the skin [80,81,82,83]. It has been demonstrated that plant extracellular vesicles increase the production in treated cells, positively contributing to regenerative processes. To better investigate this effect, fibroblasts were seeded into 24-well plates, then we induced a scratch in the cellular monolayer and analyzed collagen I expression after 48 h of treatment. As shown in Figure 7, PDEVs treatment can efficiently increase the collagen expression after 48 h, measuring about a 2-fold increase in collagen expression (5.86 ± 0.93 M.I.F.; *p* < 0.05) in PDEVs-treated cells compared to untreated control cells (3.06 ± 0.34 M.I.F.). The quantification is shown in Supplementary Figure S2a.

#### 3.4.3. PDEVs Increase MMP-9 Expression at the Wound Site

**Figure 8:**
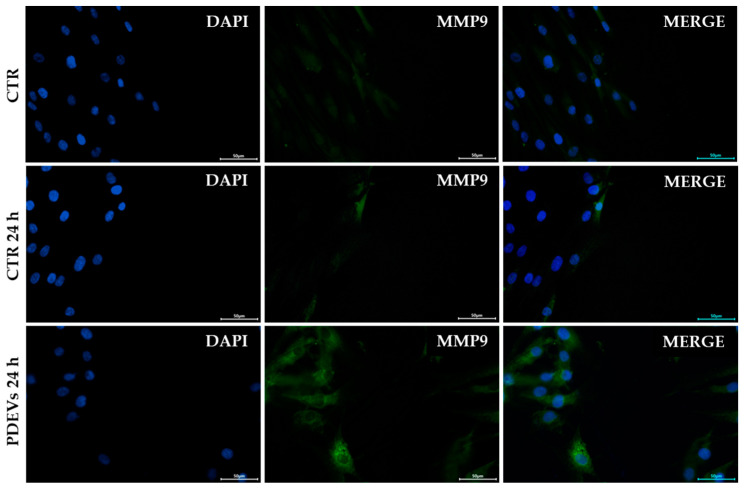
MMP-9 expression in skin fibroblasts at the wound site. Cells were stained with anti-MMP-9-FITC (green) and nuclei were counterstained with DAPI (blue). Scale bar = 50 µm.

Wound healing is a complex process involving different events, including mesenchymal cell differentiation, proliferation, migration to the wound site, and re-epithelization [41,84,85]. In this process, matrix metalloproteinases (MMPs), a family of zinc-containing enzymes, play an important role in the decomposition of extracellular matrix after injury. Among these enzymes, MMP-9 is a fundamental protein involved in cell migration and recruitment at the wound site, together with the regulation of the angiogenesis process. Moreover, while the MMP family is constitutively expressed by fibroblasts, the production of MMP-9 is induced by external stimuli, thus showing its pivotal role in tissue metabolism and regeneration [47,48,49,50,86,87,88,89,90]. In order to investigate PDEVs’ mechanism of action in skin cells, we evaluated MMP-9 expression in skin fibroblasts after the induction of an injury. Briefly, cells were seeded on coverslips in order to have a uniform monolayer, then we made a scratch and treated cells for 24 h with PDEVs. After treatment, we evaluated MMP-9 expression through immunofluorescence microscopy. Figure 8 shows images of the wound site, since it has been widely demonstrated that MMP-9 is highly overexpressed in epithelial cells at the front of the migrating epithelial sheet [47,91]. Notably, we measured a 6-fold increase in MMP-9 expression (*p* < 0.01) after 24-h treatment with PDEVs (4.73 ± 1.15 M.I.F.) compared to untreated control cells (0.73 ± 0.03 M.I.F). The quantification is shown in Supplementary Figure S2b.

#### 3.4.4. PDEVs Increase Vimentin Expression After Scratch Induction

**Figure 9:**
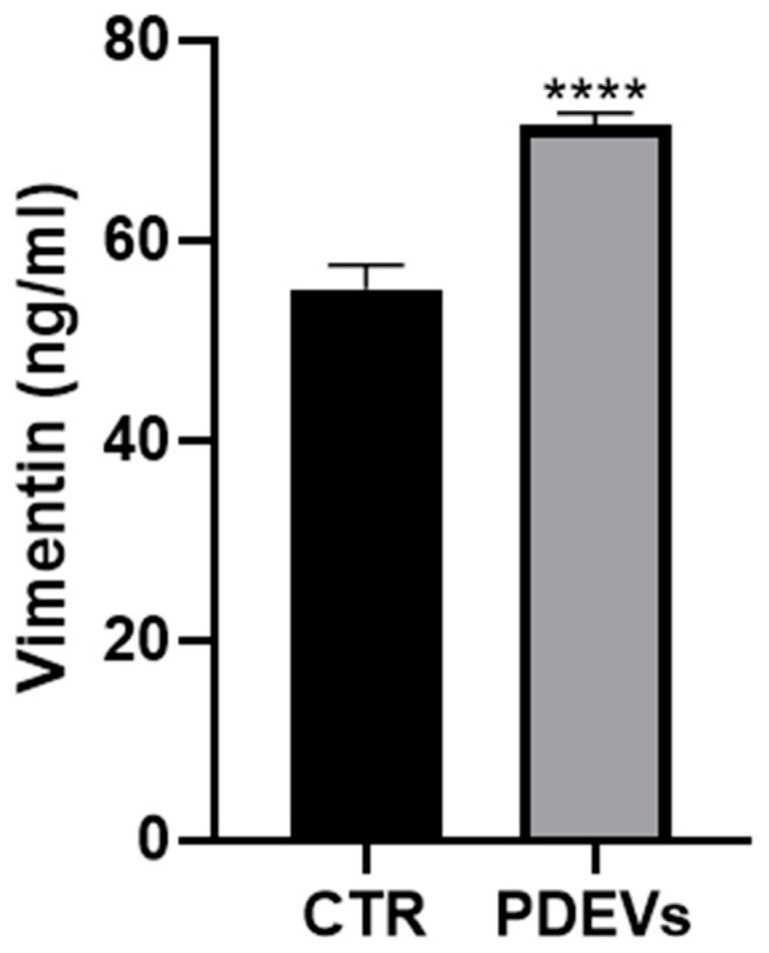
Quantification of extracellular vimentin. Data are expressed as mean ± SE. **** *p* < 0.0001. Statistical analysis was performed using unpaired *t*-test (Student’s *t*-test).

Vimentin is another key protein involved in cellular proliferation and wound repair. In fact, vimentin is an intermediate filament protein that participates in numerous processes, including cell adhesion, migration and invasion, signaling, and differentiation. In tissue repair, vimentin regulates the migration after an injury [51,52,53,54], and its concentration is directly related to the ability of cells to close a wound [92,93,94]. We thus measured vimentin concentration in cell culture supernatants after treatment with the PDEVs’ mix. Briefly, as mentioned for previous experiments, we seeded cells in order to obtain a monolayer; the fibroblasts were treated with the PDEVs’ mix for 24 h, and the concentration of vimentin was measured in the cell culture supernatant. The results showed a significant increase in vimentin in the supernatant of the PDEVs’ mix-treated cells compared to control cell cultures (Figure 9). Notably, we found a strong increase in vimentin in the PDEVs’ mix-treated human skin fibroblast cell cultures (CTR: 56 ± 2.4 ng/mL, PDEVs: 71 ± 1.3 ng/mL; *p* < 0.0001).

These results strongly support the results obtained in the wound healing experiments.

## 4. Discussion

PDEVs naturally contain a wide array of molecules, including proteins, lipids, and nucleic acids, that are all involved in the intercellular communication within the same species and between the different species and kingdoms. In fact, PDEVs contain antioxidant molecules that can moderate oxidative stress damage, scavenging ROS and enhancing the antioxidant defense systems of cells [95]. PDEVs can efficiently influence the expression of genes involved in skin health, such as regeneration, skin barrier, moisturization, and aging-related genes [96]. Taking into account these considerations, we combine different fruit extracts widely characterized to exert beneficial effects in skin regeneration and mitigation of oxidative stress damages [95,97,98,99,100,101,102,103,104,105,106,107,108]. Our previous paper and the results of this study strongly support the use of PDEVs’ mixes instead of PDEVs of single fruits to obtain the best antioxidant and anti-aging effect.

First of all, we characterized PDEVs bioactive molecules, detecting a huge amount of antioxidants, including both enzymatic bioactives, such as superoxide dismutase and catalase, and non-enzymatic ones, such as glutathione and ascorbic acid. Moreover, ATP was detected in Extracellular Vesicles, and it demonstrated not only its role as an energy source but also as signaling molecule that mediates interactions between organs and systems [109]. Consistently with other works showing that ATP positively influences skin cell proliferation, DNA repair, and collagen synthesis [94,110], we assume that ATP in PDEVs could positively exert beneficial regenerative action in combination with antioxidants naturally contained in the PDEVs. Moreover, the skin repair process consumes high amounts of energy [110,111,112]. Thus, we predict that the ATP supplementation from PDEVs could be helpful in maintaining physiological skin cell turnover, keeping cells from proliferating, and producing molecules involved in regenerative processes.

In this study, we set up a PDEVs’ mix that, while combining a heterogeneous population of extracellular vesicles in terms of size distribution and confirmed by NTA and TEM analysis, has proven highly stable in liquid suspension, also confirming previous reports [34,35,113]. Moreover, the PDEVs used in this study had a very negative zeta potential. Notably, the zeta potential value characterized the fusogenic properties of PDEVs with plasma membranes of target/recipient cells, and the more negative the zeta potential of the PDEVs, the more fusogenic they are. Based on this background, we analyzed the cellular uptake of our PDEVs’ mix by human skin fibroblasts. To this purpose and on the basis of our previous investigation [114,115], we labeled PDEVs with a fluorescent probe that specifically bound the membrane phospholipids of the extracellular vesicles. The labeled PDEVs were detectable in the target fibroblasts up to 24 h after treatment, with an increase in the fluorescence values in the following 48 and 72 h. Furthermore, after 72 h of treatment, the signal of the PDEVs overlaps with that of the nuclei, suggesting that PDEVs are able to effectively reach the genome of the target cells, possibly influencing the gene expression of recipient cells. This set of results witnessed the ability of PDEVs to be entirely uploaded into the target cells, where they fully exert their action.

Damages induced by oxidative stress can affect virtually all the cellular pathways, in turn inducing a heavy imbalance in the cellular homeostasis, of course including mitochondrial activities as well. To reproduce this condition, we treated human skin cell fibroblasts with hydrogen peroxide, which induces a redox imbalance into the cells. Following the H_2_O_2_ treatment, we first tested the ability of our PDEVs mix to revert the imbalanced mitochondria membrane potential, which is the most important indicator of mitochondrial health. The results showed that PDEVs can efficiently restore the physiological mitochondrial membrane potential after oxidation stimuli, from the first 24 h after treatment. Supporting this result, PDEVs reduce the production of mitochondrial superoxide anion in treated cells, further demonstrating their beneficial effect on mitochondrial metabolism. Also, the increase in oxidative stress damages is related to the decreased production of molecules involved in age-related processes, such as the sirtuin family of proteins. Thus, we compared the levels of extracellular sirtuin 1 before and after treatment of oxidated cells with PDEVs, showing that PDEVs increased the extracellular sirtuin levels in treated cultures, thus further supporting their skin anti-aging effect.

In a further series of experiments, we wanted to directly show the ability of our PDEV mix to influence cellular mechanisms involved in the skin regenerative processes. For this, we used the model of wound healing in cultures of human skin fibroblasts. We first showed the ability of our PDEV mix to lead to a full closure of the wound produced in the human skin fibroblast’s monolayer. Then we showed in the same model that the wound repair was associated with the increase in collagen I and MMP-9 concentrations, a protein actively involved in cellular repair mechanisms. Of interest, in our experiments, MMP-9 was more expressed in the fibroblasts’ cultures treated with PDEVs, particularly at the wound site, where the wound closure actually started. In the same cultures, we found a higher concentration of extracellular vimentin in fibroblasts treated with the PDEVs mix as compared to untreated fibroblasts. Consistent with previous reports [116], we assumed that the polyphenols contained in our PDEV mix could contribute well to the wound healing we showed in our experiments.

All-in-all, the results of our study provided scientific evidence that a mix of PDEVs from different fruits from organic farming may have a clear beneficial effect on skin aging processes. This effect passes through a clear antioxidant action due to the high levels of antioxidant molecules contained in the PDEV mix. Here we powerfully show that the PDEVs effect was due to a full uploading of the extracellular vesicles into the target cells. Our results are also supported by previous reports showing that Plant- Derived Extracellular Vesicles can efficiently penetrate recipient cells, decrease levels of oxidant species, and positively regulate regenerative processes [58,59,95,117,118,119,120,121].

## 5. Conclusions

Here we show that PDEVs isolated and concentrated from a mix of fruit extracts (grape, blood orange, tangerine, papaya, and pomegranate) contain different detectable antioxidants (superoxide dismutase, catalase, glutathione, and ascorbic acid). These vesicles are efficiently uptaken by human skin fibroblasts, where they mitigate aging processes related to oxidative damages, restoring the physiological mitochondrial function, and increasing the concentration of sirtuin 1, whose principal role is the regulation of age-related processes. Moreover, here we show that PDEVs are actively involved in maintaining the skin’s barrier function, assisting wound repair by also modulating the expression of MMP-9 and vimentin, essential proteins involved in tissue repair processes.

We believe that our results will contribute to positively changing the approach of local treatment of skin aging through the set-up of entirely new products, but implementing the local treatments of skin wounds. We can’t exclude that the local antioxidant effect of our PDEV mixes may be improved by the systemic treatment with the same mixes that have shown to burst an antioxidant reaction in the whole body [34].

## 6. Patent

The company holds a patent describing the methodology used in obtaining the mixes of the plant-derived extracellular vesicles (Logozzi M, Fais S. Nanovesicules Deriving from Biological Plants as Natural Carriers of Phyto-Complexes for Nutraceutical, Cosmetic and Regenerative Use [Internet]. 2022 [cited 1 March 2023]. Available from: https://patentscope.wipo.int/search/en/detail.jsf?docId=WO2022157726). 

## Figures and Tables

**Table 1:** Quantification of bioactive compounds in PDEVs.

Bioactive Compound	Concentration
Total Antioxidant Capacity	2.3 ± 0.2 nMol/µL
Ascorbic Acid	1910 ± 1 ng
ATP	61.3 ± 16.6 mM
Catalase	499.1 ± 2.2 mU/mL
Citric Acid	37.67 ± 1.34 µmol/L
Glutathione	11.8 ± 0.3 µM
SOD	7392.00 ± 6.03 U/mL

## Data Availability

Data is contained within the article and Supplementary Materials.

## Supplementary Materials

The following supporting information can be downloaded at: https://www.mdpi.com/article/10.3390/antiox13111373/s1, Figure S1: PDEVs treatments reduce senescence in cells; Figure S2: Increased expression of collagen I and MMP-9 after treatment with PDEVs.
