# Supplementary material for: Antioxidant Effect of a Plant-Derived Extracellular Vesicles’ Mix on Human Skin Fibroblasts: Induction of a Reparative Process
Source: Antioxidants (Basel). 2024 Nov 9;13(11):1373. doi: 10.3390/antiox13111373 (PMC11590891; doi:10.3390/antiox13111373)
Supplement: Supplementary file 1 [file antioxidants-13-01373-s001.zip › antioxidants-3172570-supplementary.pdf]

Supplementary Material

Supplementary Figure S1

PDEVs treatment reduce senescent cells

In order to mimic aging in Fibroblasts, we treated cells with hydrogen peroxide as previously described in Materials and Methods section. Then, we treated cells with PDEVs and used a staining protocol specific for senescent cells identification (CS0030, Sigma-Aldrich, USA). Briefly, cells were washed with PBS, then fixed with the appropriate solution and incubated with staining mixture in order to evaluate the percentage of cells expressing β-galactosidase (senescent cells are stained blue). As shown in Supplementary Figure S1, oxidation protocol with H_2O_2 can efficiently induce senescence in treated cells (Supplementary Figure S1b), with 17 ± 0.8 % of senescence cells compared to total cells, while in the not oxidated sample the percentage of senescent cells is 0.83 ± 0.04% (Supplementary Figure S1a). Notably, Fibroblasts oxidated with hydrogen peroxide and then treated with PDEVs for 24 h, showed a reduction of senescent cells reaching a rate of blue cells compared to total cells equal to 3.6 ± 0.2 % (Supplementary Figure S1c).

(a) (b) (c)

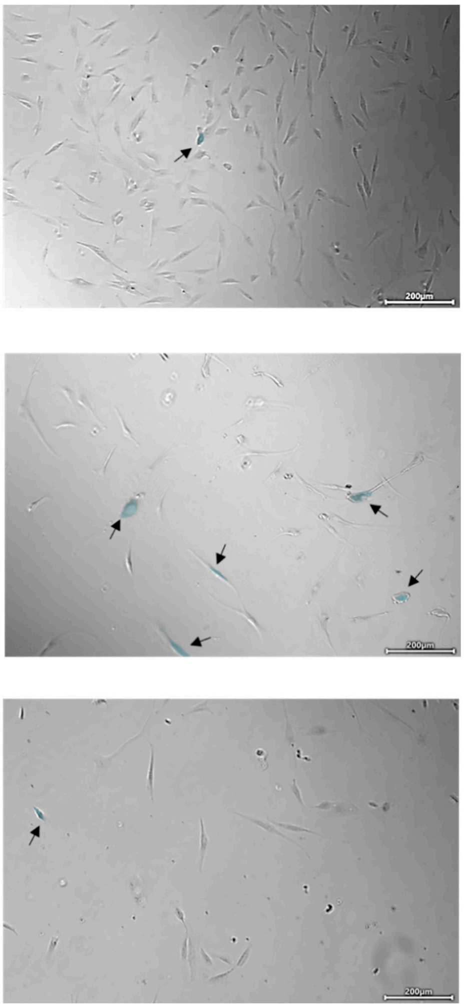

Supplementary Figure S1. Beta-galactosidase expression in Fibroblasts in (**a**) untreated control cells, (**b**) oxidated cells and (**c**) oxidated cells treated with PDEVs. Arrows indicate senescent cells. Scale bar=500µm.

Supplementary Figure S2

Increased expression of collagen I and MMP-9 after treatment with PDEVs

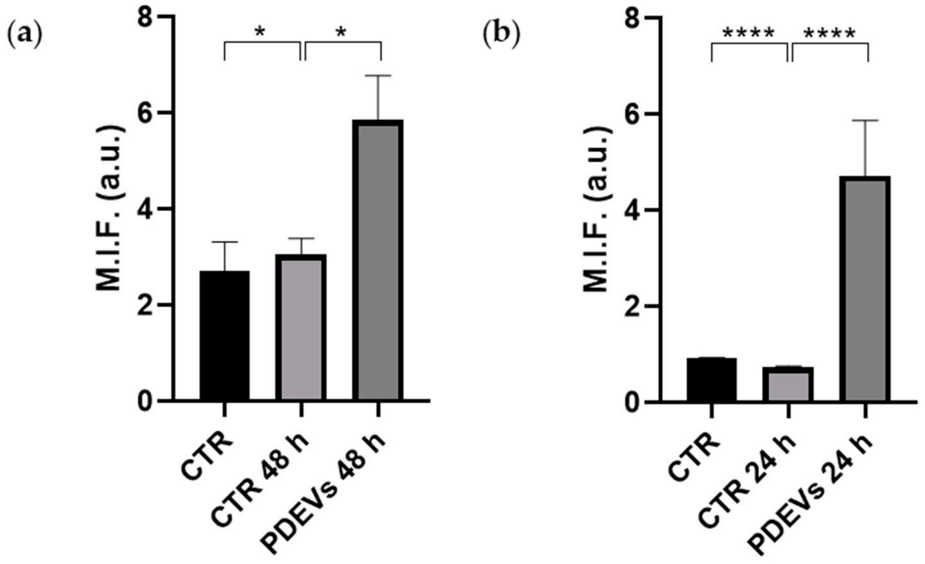

Supplementary Figure S2. PDEVs increase (**a**) Collagen I expression after 48-hour treatment and (**b**) MMP-9 expression after 24-hour treatment in *in vitro* Fibroblasts. Data are expressed as Mean Intensity of Fluorescence (arbitrary unit) ± SE. *p<0.05, ****p<0.0001. Statistical analysis was performed using one-way ANOVA Bonferroni.

As described in the Materials and Methods section, Fibroblasts treated with PDEVs were labelled with fluorescent antibodies binding collagen I and MMP-9 in order to evaluate their expression after the induction of an injury. In order to quantify the expression, the mean intensity of fluorescence of sample was measured using ImageJ software and data are summarized in Supplementary Figure S2. In cells treated with PDEVs, we measured an increased (p<0.05) collagen I fluorescence equal to 5.85 ± 0.93 M.I.F. (a.u.) after 48 hours of treatment, while in untreated control cells the fluorescence is 3.06 ± 0.34 M.I.F. (a.u.); in cells just after wound induction (CTR), the fluorescence was equal to 2.71 ± 0.61 M.I.F. (a.u.) (Supplementary Figure S2a).
Comparable to Collagen results, we measured a most abundant expression of MMP-9 in cells after 24-hour treatment with PDEVs (p<0.01), with values equal to 4.73 ± 1.15 M.I.F. (a.u.) compared to untreated control cells (0.73 ± 0.03 M.I.F. a.u.), while the fluorescence intensity for control cells at T0 was 0.93 ± 0.01 M.I.F. a.u. (Supplementary Figure S2b).
